# Demographic, clinical, and surgical features of patients undergoing thyroidectomy due to thyroid lesions in Southern Iran: A cross‐sectional study

**DOI:** 10.1002/hsr2.2012

**Published:** 2024-04-01

**Authors:** Parviz Mardani, Sepehr Koulaian, Damoun Fouladi, Fatemeh Sadat Rajaie Ramsheh, Armin Amirian, Sepehr Shahriarirad, Seyed Ali Malekhosseini, Reza Shahriarirad

**Affiliations:** ^1^ Thoracic and Vascular Surgery Research Center Shiraz University of Medical Science Shiraz Iran; ^2^ Shiraz Transplant Research Center Shiraz University of Medical Sciences Shiraz Iran; ^3^ Student Research Committee Shiraz University of Medical Sciences Shiraz Iran

**Keywords:** incidence, pathology, surgery, thyroid cancer, thyroidectomy

## Abstract

**Background and Aims:**

The incidence of thyroid cancer has witnessed a significant global increase and stands as one of the most prevalent cancers in Iran. This surge is primarily attributed to the escalating incidence of papillary thyroid cancer (PTC), with overdiagnosis emerging as an equally noteworthy factor. Consequently, this study aims to ascertain the incidence of thyroid cancer, along with its clinical presentation, demographic characteristics, and surgical features in patients undergoing thyroid surgery.

**Methods:**

This cross‐sectional study involved the evaluation of patient files from referral centers in Shiraz spanning the years 2015−2020. Demographic and clinical information pertaining to thyroid cancer was extracted and subsequently analyzed using SPSS software.

**Results:**

A total of 533 documented cases of thyroid cancer undergoing surgery revealed an annual rate of 89 cases in our location. The average age of the patients was 43.9 ± 13.4 years (ranging from 13 to 92), with females constituting 429 (83.5%) of the cases, and 278 (54.1%) being malignant. Conventional PTC emerged as the most prevalent pathology, accounting for 239 (45.0%) of the cases. Patients with thyromegaly exhibited significantly higher incidences of nonmalignant tumors (*p* = 0.01), while those with malignant tumors were notably younger than those with nonmalignant tumors (*p* = 0.001).

**Conclusion:**

Our study revealed a progressive rise in the number of patients undergoing thyroidectomy over the years, with PTC constituting the majority of cases. Malignant cases were more frequently observed in younger patients, and in smaller lesion sizes, highlighting the importance of early screening and optimizing detection methods, especially in high‐risk populations.

## 1 BACKGROUND

There has been a significant rise in the number of individuals diagnosed with thyroid cancer over the last 30 years in the United States and other developed countries.^1^, ^2^ While some researchers attribute this increase to a genuine rise in thyroid cancer cases, others contend that the heightened incidence is a result of improved diagnostic testing, such as ultrasonography (US) and fine‐needle aspiration (FNA) biopsy, leading to enhanced disease detection.^3^, ^4^, ^5^, ^6^

The four common subtypes of thyroid cancer are papillary carcinomas (80%), follicular carcinomas (10%), medullary thyroid carcinomas (5%−10%), and anaplastic carcinomas (1%−2%).^7^, ^8^, ^9^ The majority of thyroid cancers, such as papillary thyroid cancer (PTC), follicular thyroid cancer (FTC), poorly differentiated thyroid cancer, and anaplastic thyroid cancer (ATC), originate from follicular thyroid cells. While many authors categorize papillary and follicular carcinomas together as differentiated carcinomas, they may occasionally exhibit rapid growth instead of the typical slow progression.^10^, ^11^, ^12^, ^13^, ^14^ Over 90% of thyroid nodules are benign lesions that will never become clinically significant.^15^ However, in some cases, whether palpable or non‐palpable, they have the potential to be malignant.^16^ The 2015 ATA guidelines recommend total thyroidectomy in low‐ to intermediate‐risk patients.^17^ As per the guidelines defined boundaries for central lymph node dissection (CLND), level VII station lymph nodes (LN) should be included in CLND. However, the routine performance of thymectomy for this purpose remains unclear.^17^, ^18^, ^19^ A comprehensive history and physical examination, laboratory techniques, neck US, and at times FNA are essential for distinguishing lesions with a low likelihood of malignancy from those that are malignant.

In Iran, thyroid cancer is the seventh most common cancer in females and the 11th most common cancer in males, in which its incidence varies among different provinces.^20^, ^21^ Fars province reported more than 10 incidences of thyroid cancer per 100,000 in 2010, which, compared to 1990–2001, demonstrates a noticeable increase from 0 to 1 per 100,000.^22^ Given the high volume of patients undergoing thyroidectomy at our referral center, we designed this retrospective study to evaluate the incidence, demographic characteristics, clinical presentation, and surgical features of patients undergoing thyroidectomy due to thyroid lesions. Our objective also includes reporting the prevalence of various thyroid pathologies in our study location and assessing the association between the patients' features and their pathology reports to identify potential risk groups for thyroid malignancies.

## 2 METHODS

In this retrospective cross‐sectional study, we evaluated the medical records of patients admitted to Namazi and Bu‐ali Hospitals in Fars province from 2015 to 2020. These hospitals serve as referral centers for thyroid surgery in Southern Iran. We included patients with postoperative sample pathological reports and excluded any files related to pathologies unrelated to the thyroid.

Two qualified general practitioners carefully reviewed the files by referring to the hospital archives and categorizing them according to specific disease codes. From these patient records, information pertaining to thyroid cancer disease, including baseline demographic, clinical, operative, and pathologic features was extracted. Demographic data included age (categorized into groups of ≤18, 19–25, 26–35, 36–45, 46–55, >55) and gender. Clinical features included signs and symptoms on admission, as well as a history of previous subtotal thyroidectomy. Surgical features comprised information on thyroidectomy, LN dissection, thymectomy, the number of isolated and involved nodes (categorized as none, 1−10, 11−20, >20, and none, 1–3, >3, respectively), size, area, and volume of the tumoral lesion (categorized as ≤1, 1–3, 3.01–5, >5 cm for size, ≤1, 1–10, >10 cm^2^ for area, and ≤1, 1−10, >10 cm^3^ for volume), TNM classification,^23^ location of the lesion, focality, capsule involvement, mitosis, extrathyroidal invasion, lymphovascular invasion (LVI), perineural invasion, encapsulation, calcification, marginal involvement, and necrosis. The pathology features were assessed based on postoperation permanent pathology samples.

The data were inputted into SPSS version 26.0 software and subsequently analyzed. Descriptive statistics are presented as frequency and percentage (%). The distribution of continuous variables was assessed using the Shapiro−Wilk test. For normally distributed variables, the results are reported as mean and standard deviation (SD), while for non‐normally distributed variables, the values are reported as median and the first and third quantiles [Q1–Q3]. The association between thyroid cancer features and related factors is examined using specific *χ* ^2^ tests or Fisher's exact test for categorical variables, and the independent sample *t*‐test or Mann−Whitney test for continuous variables. A *p*‐value less than 0.05 is considered statistically significant.

## 3 RESULTS

During the study period, a total of 533 thyroid cancer cases that underwent surgery were documented, indicating an average annual rate of 88.8 cases. Among these patients, 19 were excluded due to an incomplete final pathology report. The remaining 514 cases were included in our analysis. The average age of the patients was 43.9 ± 13.4 years, ranging from 13 to 92, with 429 (83.5%) of them being females. In our study, 278 cases (54.1%) were identified as malignant, while 236 cases (45.9%) were nonmalignant. Table 1 illustrates the baseline features of the patients in our study. The analysis revealed that gender and a previous history of subtotal thyroidectomy showed no significant association with thyroid malignancy. However, patients with thyromegaly exhibited significantly higher incidences of nonmalignant tumors (*p* = 0.01). Additionally, patients with malignant tumors were significantly younger compared to those with nonmalignant tumors (*p* = 0.001).

**Table 1:** Demographic and clinical features of thyroidectomy patients in southern Iran.

Variable		Value (*N* = 514)	Histopathology		*p* Value^a^
			Malignant (*n* = 278)	Nonmalignant (*n* = 236)	
Age (years); mean ± standard deviation		43.9 ± 13.4	42.0 ± 13.9	46.0 ± 12.6	**<0.001**
Age group; *n* (%)	≤18	5 (1.0)	4 (1.4)	1 (0.4)	**0.003**
	19–25	27 (5.3)	17 (6.1)	10 (4.2)	
	26–35	127 (24.7)	85 (30.6)	42 (17.8)	
	36–45	137 (26.7)	74 (26.6)	63 (26.7)	
	46–55	120 (23.3)	55 (19.8)	65 (27.5)	
	>55	98 (19.1)	43 (15.5)	55 (23.3)	
Gender; *n* (%)	Male	85 (16.5)	48 (17.3)	37 (15.7)	0.63
	Female	429 (83.5)	230 (82.7)	199 (84.3)	
Sign and symptoms; *n* (%)	Thyromegaly	35 (6.8)	16 (26.2)	19 (51.4)	**0.01**
	Mass lesion	29 (5.6)	22 (36.1)	7 (18.9)	0.07
	Dyspnea	18 (3.5)	10 (16.4)	8 (21.6)	0.59
	Dysphagia	11 (2.1)	6 (9.8)	5 (13.5)	0.74
	Hoarseness	9 (1.7)	7 (11.5)	2 (5.4)	0.48
	Weight loss	3 (0.6)	2 (3.3)	1 (2.7)	1.00
	Neck pain	3 (0.6)	2 (3.3)	1 (2.7)	1.00
	Lymphadenopathy	4 (0.8)	4 (6.6)	0 (0)	0.29
	Weakness	1 (0.2)	1 (1.6)	0 (0)	1.00
	Generalized edema	1 (0.2)	1 (1.6)	0 (0)	1.00
	Dry mouth	1 (0.2)	1 (1.6)	0 (0)	1.00
	Chest pain	1 (0.2)	1 (1.6)	0 (0)	1.00
Previous subtotal thyroidectomy		10 (2%)	5 (2.0)	5 (2.1)	1.00

*Note*: Bold values indicate a significant association.

^a^ *χ* ^2^/Fisher's exact test or independent sample *t*‐test.

Table 2 illustrates the clinical and surgical features of the patients and their association with tumor malignancy. Total thyroidectomy (92.1%, *p* < 0.001) and CLND (29.8%, *p* = 0.005) were the most frequently performed surgical methods in our study, and both were significantly associated with tumor malignancy. Among the majority of the patients, no nodules were involved (58.9%), and an involved/resected LN ratio of 97.04% (197/203) was observed. The average size of the largest tumor diameter was 2.1 ± 1.78 cm, ranging from 0.1 to 14 cm.

**Table 2:** Clinical and surgical features of patients who underwent thyroidectomy.

Variable		Value (*N* = 514)	Histopathology		*p* Value^a^
			Malignant (*n* = 278)	Nonmalignant (*n* = 236)	
Thyroidectomy	Total	478 (93.0)	269 (96.8)	208 (88.6)	**<0.001**
	Subtotal	23 (4.5)	5 (1.8)	18 (7.6)	**0.001**
	Near total	3 (0.6)	1 (0.4)	2 (0.8)	0.60
	Retrosternal component	2 (0.8)	0 (0)	2 (0.8)	0.21
	Unilateral lobectomy	9 (1.8)	3 (1.1)	6 (2.5)	0.31
	Ischiotomy	6 (1.2)	2 (0.7)	4 (1.7)	0.42
Thymectomy		7 (1.4)	5 (2.0)	2 (0.9)	0.45
Lymph node dissection	Central	159 (71.9)	112 (65.5)	47 (94.0)	**0.005**
	Central and unilateral	28 (12.7)	27 (15.8)	1 (2.0)	
	Central and bilateral	19 (8.6)	17 (9.9)	2 (4.0)	
	Unilateral	8 (1.6)	8 (2.9)	0 (0)	
	Bilateral	4 (0.8)	4 (1.4)	0 (0)	
	Radical	3 (0.6)	3 (1.1)	0 (0)	
Isolated nodule	Median [Q1–Q3]	9 [4–17]	9 [4–17]	7 [2–18.3]	0.71
	None	8 (3.9)	6 (3.1)	2 (16.7)	0.16
	1–10	104 (51.2)	98 (51.3)	6 (50.0)	
	11–20	56 (27.6)	54 (28.3)	2 (16.7)	
	>20	35 (17.2)	33 (17.3)	2 (16.7)	
Involved nodule	Median [Q1–Q3]	0 [0–2]	2 [0–10]	0 [0–0]	0.08
	None	116 (58.9)	107 (57.2)	9 (90.0)	0.09
	1–3	49 (24.9)	49 (26.2)	0 (0)	
	>3	32 (16.2)	31 (16.6)	1 (10.0)	
Size; median [Q1–Q3]	Largest diameter; (cm)	1.5 [1–3]	1.2 [0.8–2]	3.0 [2.0–4.0]	**<0.001**
	Area (cm^2^)	1.77 [0.57–4.91]	1.02 [0.39–3.04]	5.30 [2.59–9.42]	**<0.001**
	Volume (cm^3^)	0.88 [0.14–4.71]	0.47 [0.10–1.98]	5.00 [1.60–12.22]	**<0.001**
Largest size group (cm)	≤1	113 (35.6)	102 (43.8)	11 (13.1)	**<0.001**
	1−3	153 (48.3)	113 (48.5)	40 (47.6)	
	3.01−5	33 (10.4)	11 (4.7)	22 (26.2)	
	Above 5	18 (5.7)	7 (3.0)	11 (13.1)	
Area group (cm^2^)	≤1	117 (39.1)	106 (48,8)	11 (13.4)	**<0.001**
	1−10	155 (51.8)	99 (45.6)	56 (68.3)	
	Above 10	27 (9.0)	12 (5.5)	15 (18.3)	
Volume group (cm^3^)	≤1	126 (51.4)	116 (62.7)	10 (16.7)	**<0.001**
	1−10	86 (35.1)	55 (29.7)	31 (51.7)	
	Above 10	33 (13.5)	14 (7.6)	19 (31.7)	
Tumor stage	T1/2	278 (87.7)	203(75.2)	67(24.8)	**<0.001**
	T3/4	39 (12.3)	14 (6.0)	25 (29.8)	
Location	Bilateral	83 (25.1)	44 (18.5)	39 (41.9)	**<0.001**
	Bilateral and isthmus	23 (6.9)	14 (5.9)	9 (9.7)	
	Diffuse	1 (0.4)	1 (0.3)	0 (0)	
	Isthmus	8 (2.4)	7 (2.9)	1 (1.1)	
	Unilateral	185 (55.9)	143 (60.1)	42 (45.2)	
	Unilateral and isthmus	31 (9.4)	29 (12.2)	2 (2.2)	
Focality	Unilateral	128 (52.9)	121 (52.4)	7 (63.6)	0.47
	Bilateral	114 (47.1)	110 (47.6)	4 (36.4)	
Thyroid capsule		43 (18.7)	42 (19.8)	1 (5.6)	0.21
Mitosis	Without mitosis	167 (72.0)	159 (71.6)	8 (80.0)	1.00
	One mitosis	49 (21.1)	47 (21.2)	2 (20.0)	
	Above one	16 (6.9)	16 (7.2)	0 (0)	
Extrathyroidal extension		25 (10.3)	24 (10.3)	1 (10.0)	1.00
Lymphovascular invasion		48 (18.8)	47 (19.7)	1 (5.9)	0.21
Perineural invasion		9 (3.8)	8 (3.5)	1 (11.1)	0.30
Encapsulation	Not seen	155 (65.1)	149 (65.4)	6 (60.0)	0.83
	Seen	72 (30.3)	68 (29.8)	4 (40.0)	
	Partial or not complete	11 (4.6)	11 (4.8)	0 (0)	
Calcification		117 (53.7)	115 (54.2)	2 (33.3)	0.42
Resected margin	Free	219 (95.2)	207 (95.4)	12 (92.3)	0.48
	Involved	11 (4.8)	10 (4.6)	1 (7.7)	
Necrosis	Positive	3 (10.7)	2 (7.4)	1 (100)	0.11
	Negative	25 (89.3)	25 (92.6)	0 (0)	

*Note*: Bold values indicate a significant association.

^a^ *χ* ^2^/Fisher's exact test or Mann−Whitney *U t*‐test.

Malignant pathologies more frequently underwent total thyroidectomy (*p* < 0.001; OR: 3.86; 95% CI: 1.78−8.40), while subtotal thyroidectomy was more frequently carried out in nonmalignant pathologies (*p* = 0.001; OR: 4.51; 95% CI: 1.65–12.34). Malignant tumors were significantly smaller in size, area, and volume compared to nonmalignant tumors. Lesions measuring ≤1 cm in largest diameter were strongly correlated with malignant histopathology (90.4%), while lesions with a largest diameter of above 5 cm were mostly seen in nonmalignant cases (61.1%). The majority of malignant lesions were located unilaterally (51.79%; *p* < 0.001). Also, 83 (15.63%) patients showed signs of LN involvement, and two patients (0.003%) had distant metastasis.

The results of pathological evaluation of the resected lesions are presented in Table 3. The most frequently observed pathology was PTC in 239 (45%) of the cases, followed by MNG at 31.1%, Hashimoto's thyroiditis at 4.6%, and the follicular variant of PTC with capsular vascular invasion at 4.3%. The malignant group primarily comprised differentiated thyroid cancer, with ATC observed in only 5 cases (1%) within the undifferentiated subgroup. FTC and Hürthle cell carcinoma were identified in 6 (1.2%) and 4 (0.8%) cases, respectively, while MTC constituted 6 cases (1.1%). In the nonmalignant group, follicular lesions, including MNG at 31.1%, Hashimoto's thyroiditis at 4.6%, and colloid goiters at 3.6%, constituted the primary findings. Other nonmalignant findings, such as lymphocytic thyroiditis (2.3%) and Graves' disease (1.1%) were observed in a smaller number of cases.

**Table 3:** Histopathological findings from thyroidectomy patients in Southern Iran.

Group	Subgroup			Frequency (%)
Malignancy	Total			279 (54.2)
	Differentiated	Derived from follicular epithelioma	PTC conventional	239 (45.0)
			FTC	6 (1.2)
			Hurthle cell carcinoma	4 (0.8)
			PTC follicular variant with capsular vascular invasion	23 (4.3)
		Derived from parafollicular epithelioma	MTC	6 (1.1)
	Undifferentiated	Anaplastic thyroid cancer		5 (1)
	Differentiated + undifferentiated (synchronous thyroid tumors)	Anaplastic and Hurthle cell		0 (0)
		Anaplastic and papillary		1 (0.2)
	Primary thyroid lymphoma	Non‐Hodgkin		0 (0)
	Primary thyroid sarcoma			0 (0)
Nonmalignant	Total			236 (45.8)
	Follicular lesion	NIFTP		5 (0.9)
		Nodular goiter		
		Colloid goiter		19 (3.6)
		Follicular adenoma		10 (1.9)
		MNG		165 (31.1)
		Hashimoto		24(4.6)
		Hurthle cell adenoma		4 (0.8)
		Follicular hyperplasia		11 (2.2)
		Adenomatous goiter		5 (0.9)
	Lymphocytic thyroiditis			12 (2.3)
	Squamous metaplasia			1 (0.2)
	Diffuse hyperplasia			2 (0.4)
	Cystic fluid			1 (0.2)
	Graves			6 (1.1)
Both malignant and nonmalignant	PTC and follicular hyperplasia			2 (0.4)
	PTC and MNG			2 (0.4)
	Hashimoto and Hurthle cell carcinoma			2 (0.4)
Nonthyroidal	Parathyroid adenoma	Nonmalignant		2 (0.4)
	Metastatic lung carcinoma	Malignant		1 (0.2)
	Benign thymoma	Nonmalignant		1 (0.2)
Unclear	Follicular neoplasm			7 (1.3)
	Hurthle cell change			1 (0.2)
	Lymphangioma			1 (0.2)
	Thymoma			1 (0.2)
	FLUS			2 (0.4)

Abbreviations: FLUS, follicular lesion of undetermined significance; FTC, follicular thyroid cancer; MNG, multinodular goiter; MTC, medullary thyroid carcinoma; NIFTP, noninvasive follicular thyroid neoplasm with papillary‐like nuclear features; PTC, papillary thyroid cancer.

Furthermore, in the unclear or suspicious group, follicular neoplasm emerged as the most common diagnosis. Some non‐thyroidal masses were also identified, including nonmalignant parathyroid adenoma (two cases), malignant metastatic lung cancer (one case), and benign thymoma (one case).

## 4 DISCUSSION

In this cross‐sectional study, we observed an average annual rate of 88.8 cases of thyroid cancer undergoing thyroidectomy in our location, with PTCs being the predominant histopathological finding. Notably, this study represents the first population‐level investigation examining the impact of patient demographics and clinical and pathological factors on thyroidectomy in patients with thyroid lesions in Iran. Our findings indicate that age, thyromegaly, method of thyroidectomy and LN dissection, size, and location of thyroid lesions were correlated with tumor malignancy. As the incidence of thyroid cancer continues to steadily rise, an evolving understanding of the demographic features involved, as well as clinical and histopathological characteristics of these lesions, is necessary to improve approaches to evaluation and management and to refine current healthcare policies.^24^

In our study, a higher age was associated with a lower rate of malignant tumors compared to nonmalignant tumors. Additionally, age groups 26−35 and 46−55 constituted the majority of cases of malignant (30.6%) and nonmalignant (27.5%) tumors in each category, respectively. In a study by Do et al., which evaluated the association of age with the risk of malignancy in 1022 patients undergoing consecutive thyroidectomy, rates of DTC did not differ between patients aged <45 or ≥45, and it occurred more frequently in patients younger than 50 years.^25^ Kwong et al. also found that with increasing age, the risk of thyroid nodules being malignant decreases. However, clinically relevant nodules are more prevalent in older age and carry a higher risk of manifesting as a more severe histological phenotype.^26^ Moreover, a study evaluating the demographic characteristics of thyroid cancer over the last two decades found the highest rate of increase in this type of cancer to be in the age range of 30−50, with PTC exhibiting the highest rate of increase, followed by FTC.^27^ Considering that the onset age of thyroid cancer is trending younger in recent decades,^28^ early preventive and diagnostic methods, including screening, are suggested for the population in the age group at higher risk.

The majority of patients in our study were female (83.5%). The higher incidence of benign thyroid disease in women is often thought to lead to more frequent thyroid investigations and surgeries. However, in a study by Machens et al.,^29^ which primarily featured gross thyroid tumors, there wasn't a significant difference in the prevalence of sporadic thyroid microcarcinomas between men and women. This suggests that differential screening may not be a major factor in the observed gender disparities in sporadic thyroid cancers. Another potential explanation could be gender‐specific behavioral differences, with men tending to seek medical attention later than women. Due to the retrospective nature of our study and a high referral rate, no direct data on patients' delay were available. If gender‐specific delay was a significant factor, it might have contributed to the generally larger overall tumor size, particularly in FTC.^30^ The higher prevalence of Graves' disease among women could also be a contributing factor to the observed sex differences in our study.^31^ Several factors may contribute to these sex differences, including the higher incidence of thyroid carcinoma and LN metastasis in men, necessitating more intricate or extensive surgeries.^32^, ^33^ Studies also propose that men may have tougher neck tissues and stronger neck muscles, leading to the potential slipping of ligatures or reopening of previously tied vessels, thereby increasing the risk of hematoma.^33^, ^34^ Additionally, men are more prone to conditions such as hypertension and engage in unfavorable habits like smoking and drinking.^34^ However, these factors were not extensively evaluated in our study. Another aspect to consider is the documented higher susceptibility of male patients to postoperative neck hematoma following total thyroidectomy.^35^, ^36^ Neck hematoma is recognized as the primary cause for reintervention post‐thyroidectomy.^37^ Further exploration is needed to determine if anatomical distinctions could account for this observation.^37^ Despite potential variations in disease rates between genders, this does not necessarily imply distinct sex‐specific rates of malignant progression to thyroid cancer, as our results also demonstrated no significant difference in the incidence of malignancy between male and female gender.

Although we did not examine the socioeconomic status of the patients in our study, previous research has emphasized the persistent challenges of socioeconomic disparities in healthcare access and health beliefs. Individuals with underinsured or uninsured status tend to bear a greater burden of benign thyroid diseases during thyroidectomy, as evidenced by larger pathologic thyroid volumes.^38^

In our study, the most common presentation of thyroid cancer was thyromegaly. In the malignant group, mass lesions were found to be the predominant clinical manifestation; however, the development of this symptom was not significantly associated with the incidence of thyroid cancer. While only 10% of thyroid nodules are larger than average, these lesions could potentially become clinically significant tumors.^15^, ^39^ In our case, lesions greater than 5 cm in the largest size were observed in 18 (5.6%) patients, of which 61.1% were classified as nonmalignant. In contrast, of the 114 patients who had lesions smaller than 1 cm, 90.4% were revealed to have malignant tumors. While these findings suggest that larger thyroid lesions, whether cancerous or noncancerous, are negatively associated with malignant tumors, several studies find size to have no significant relationship with tumor malignancy,^40^, ^41^, ^42^ and others associate the increase in tumor size with higher chances of malignancy.^43^, ^44^ Thus, thyromegaly alone, without considering the pathological aspect of existing lesions, can be misleading in terms of distinguishing between low‐ and high‐risk patients.

To date, available data regarding the clinical significance and prognostic role of LVI remain controversial. In a study by Sezer et al., which evaluated over 600 patients with PTC, the authors reported a correlation between LVI and several factors, including age, sex, tumor size, metastatic LNs, extrathyroidal extension, perineural invasion, and capsular invasion, considering LVI as a marker of aggressive thyroid cancer.^45^ Associations between LVI and LN metastasis, as well as distant metastasis, have also been reported.^46^, ^47^ Major vascular invasion is considered to have a significant impact on the survival of patients with thyroid cancer by potentially differentiating between benign and malignant tumors.^48^ In a study of 45,415 patients with PTC by Pontius et al., compromised survival was observed in patients with LVI compared to those without LVI, suggesting a higher risk of mortality in patients with PTC undergoing thyroidectomy.^47^ However, our study did not show any significant correlation between LVI and tumor malignancy.

Regional LN metastasis is a variable that reflects the extent of a tumor. Over the years, the prognostic value of LN ratio has been studied, indicating its significant role in predicting the recurrence of PTC^49^ and determining the outcome of patients with MTC.^50^ While an optimal cut‐off value of 0.50 has been suggested for patients with PTC undergoing total LN dissection,^51^ our study revealed an involved/resected LN ratio of 97.04%, encompassing all subtypes of thyroid cancer. These findings highlight the necessity for further evaluation of the effects of LN ratio on patient outcomes, particularly in less common subtypes of thyroid cancer where available data is scarce.

In the current study, over half of the patients undergoing thyroidectomy had malignant tumors. According to pathology results, PTC was the most common type (49.3%), and among those with nonmalignant masses, 165 (31.1%) cases were diagnosed with MNG. FTC, MTC, and ATC were seen in 1.2%, 1.1%, and 1% of the patients, respectively. In a 10‐year retrospective study by Alnuaimi et al., PTC was the most common thyroid cancer (194 cases or 95.6%), followed by FTC (2%), MTC (1.5%), and medullary‐papillary carcinoma (0.9%).^52^ Reviewing the findings of our study, atypia of undetermined significance/follicular lesion of undetermined significance (AUS/FLUS) (containing MNG and Hashimoto) was the most common type in patients with benign cancer. Additionally, similar to global rates, MNG cases were far more frequent than Hashimoto's.^53^, ^54^, ^55^ Also, the frequencies of benign macrofollicular lesions, including adenomatous goiter, benign follicular nodule, and colloid goiter, were lower than MNG. According to a population‐based study by Miranda‐Filho et al. based on the data collected from 25 countries during 2008−2012, in addition to PTC being responsible for the overall rise in thyroid cancer, FTC and MTC did not show consistent patterns in different countries, and a slight decrease in ATC was seen.^56^ Therefore, it is recommended that more regional studies be performed to determine possible risk factors for less common subtypes of thyroid cancer.

On a similar note, the rise in thyroid cancer incidence can be largely attributed to overdiagnosis. This issue is evident in a study in South Korea, where a 35% reduction in cases of thyroidectomy was observed after a high‐profile media appeal recommended against thyroid screening with ultrasound.^57^ In this regard, in 2017, the United States Preventive Services Taskforce recommended against thyroid cancer screening in asymptomatic patients, emphasizing that screening in this population has no net benefit or might even induce more harm than good.^58^ An instance of potential harm can be seen in the high percentage of total thyroidectomies in patients with thyroid cancer (85% in a study by L. Davies, as well as 92.1% in our study), which can put patients at risk of postoperative complications.^2^ Considering that the limited variations of thyroid cancer that can cause mortality almost always present with symptoms, even if these subtypes are detected by screening, the course of their outcomes is unlikely to be affected.^59^, ^60^

In our study, a total of 478 (93.0) total thyroidectomies and 23 (4.5) subtotal thyroidectomies were performed, which with the 2015 ATA guidelines.^17^ Studies support the notion that total thyroidectomy is associated with better overall recurrence‐free survival compared to lobectomy.^61^, ^62^, ^63^ In the context of thyroid cancer, the management of regional cervical LNs has been a subject of considerable debate.^64^, ^65^, ^66^, ^67^, ^68^, ^69^, ^70^ Approximately one‐third of thyroid cancer patients present with regional nodal disease,^71^, ^72^ and the clinical approach to LN dissection may vary based on the underlying pathology.^73^ In our study, total thyroidectomy along with CLND was performed in 29.8% of the patients, 70.4% of whom were associated with a malignant thyroid lesion. Several studies have also highlighted potential complications of CLND, including hypoparathyroidism, damage to the superior branch of the recurrent laryngeal nerve, and damage to the trachea or esophagus.^74^, ^75^ These complications seem to increase when central neck dissection is performed after total thyroidectomy.^76^ Regarding the thymus, limited cases in our study underwent thymectomy, with no significant correlation found with malignant tumor occurrence. Studies have stated that routine thymectomy offers minimal oncologic benefit and poses a high risk of postoperative hypocalcemia.^18^, ^77^ Li et al. advocated for thymus preservation, citing its benefits in safeguarding parathyroid glands and preventing transient hypoparathyroidism.^78^ Nasiri et al. reported a higher incidence of hypocalcemia in PTC patients undergoing CLND with thymectomy, recommending against this procedure.^79^ Overall, evidence suggests limited oncologic benefits and increased risks associated with thymectomy during CLND. Nonetheless, specialized randomized studies and long‐term follow‐ups are highly necessary to provide precise answers regarding the prophylactic and therapeutic to what extent of surgical resection can be beneficial for patients undergoing thyroidectomy.

Ultimately, while PTC has been the most common histopathological finding in thyroid cancer studies, the focus on this entity has limited the exploration of other less‐common types of thyroid cancer and their association with demographic and tumor clinical characteristics. This study addresses this gap, leveraging advanced diagnostic tools, updated medical diagnostic methods, and expert physicians as strengths. Additionally, the study provides a valuable overview of various types of both malignant and nonmalignant thyroid cancers in correlation with demographic and clinical findings. It is recommended that more detailed studies be conducted to evaluate risk factors, tumor characteristics, treatment options, and the survival and mortality risks of different types of thyroid tumors, considering the significant findings presented here.

However, the study has limitations. The lack of a long follow‐up period and the absence of an analysis of socioeconomic and racial factors are notable limitations. Since the study is based on thyroid cancer patients who underwent thyroidectomy, not all existing cases of thyroid cancer were included, and the amount of missing data, along with the lack of information regarding long‐term follow‐up, adds to the study's limitations.

## 5 CONCLUSIONS

Our findings indicate an annual rate of 88.8 thyroidectomies in Southern Iran, with a predominant prevalence of PTC. Key associations with tumor malignancy include age, thyromegaly, total thyroidectomy, and LN dissection. Demographic trends reveal that higher age is linked to a lower rate of malignant tumors, consistent with existing literature. Our study offers crucial insights into the thyroid cancer status in Southern Iran, highlighting the need for further research to explore risk factors, treatment outcomes, and long‐term prognosis for various thyroid tumor types, and studies consisting of all operated and non‐operated patients with thyroid cancers.

## AUTHOR CONTRIBUTIONS

**Parviz Mardani**: Conceptualization; investigation; supervision. **Sepehr Koulaian**: Data curation; writing—original draft. **Damoun Fouladi**: Supervision; validation; writing—review and editing. **Fatemeh Sadat Rajaie Ramsheh**: Writing—original draft. **Armin Amirian**: Investigation; validation. **Sepehr Shahriarirad**: Writing—original draft. **Seyed Ali Malekhosseini**: Conceptualization; resources; validation. **Reza Shahriarirad**: Conceptualization; formal analysis; investigation; project administration; software; validation; writing—original draft; writing—review and editing.

## CONFLICT OF INTEREST STATEMENT

The authors declare no conflict of interest.

## ETHICS STATEMENT

The ethics committee of Shiraz University of Medical Sciences approved this study (IR.SUMS.MED.REC.1400.349). Patients' information was deidentified before data analysis and confidentiality of patient information was guaranteed and protected. Based on the retrospective nature of our study, written informed consent was waived by the Ethics committee of Shiraz University of Medical Sciences, and their information was obtained from their hospital records. Permission to carry out the study and access patient records was sought from the Shiraz University of Medical Science administrators, and the study was conducted in compliance in accordance with the relevant guidelines and regulations and the Declaration of Helsinki and was also approved by the ethics committee of the university.

## TRANSPARENCY STATEMENT

The lead author Reza Shahriarirad affirms that this manuscript is an honest, accurate, and transparent account of the study being reported; that no important aspects of the study have been omitted; and that any discrepancies from the study as planned (and, if relevant, registered) have been explained.

## Data Availability

SPSS data of the participant can be requested from the authors. Please write to the corresponding author if you are interested in such data.

## References

[1] Davies L , Ouellette M , Hunter M , Welch HG . The increasing incidence of small thyroid cancers: where are the cases coming from? Laryngoscope. 2010;120(12):2446‐2451. 21108428 10.1002/lary.21076

[2] Davies L , Welch HG . Current thyroid cancer trends in the United States. JAMA Otolaryngol‐Head Neck Surg. 2014;140(4):317‐322. 24557566 10.1001/jamaoto.2014.1

[3] Brito JP , Morris JC , Montori VM . Thyroid cancer: zealous imaging has increased detection and treatment of low risk tumours. BMJ. 2013;347:f4706. 23982465 10.1136/bmj.f4706

[4] Udelsman R , Zhang Y . The epidemic of thyroid cancer in the United States: the role of endocrinologists and ultrasounds. Thyroid. 2014;24(3):472‐479. 23937391 10.1089/thy.2013.0257 PMC3949447

[5] How J , Tabah R . Explaining the increasing incidence of differentiated thyroid cancer. Can Med Assoc J. 2007;177(11):1383‐1384. 18025430 10.1503/cmaj.071464 PMC2072976

[6] Zhang Y , Zhu Y , Risch HA . Changing incidence of thyroid cancer. JAMA. 2006;296(11):1350. 10.1001/jama.296.11.1350-a 16985224

[7] Haugen BR . 2015 American Thyroid Association management guidelines for adult patients with thyroid nodules and differentiated thyroid cancer: what is new and what has changed? Cancer. 2017;123(3):372‐381. 27741354 10.1002/cncr.30360

[8] Ezaki H , Ebihara S , Fujimoto Y , et al. Analysis of thyroid carcinoma based on material registered in Japan during 1977‐1986 with special reference to predominance of papillary type. Cancer. 1992;70(4):808‐814. 1643612 10.1002/1097-0142(19920815)70:4<808::aid-cncr2820700415>3.0.co;2-l

[9] Cady B , Sedgwick CE , Meissner WA , Wool MS , Salzman FA , Werber J . Risk factor analysis in differentiated thyroid cancer. Cancer. 1979;43(3):810‐820. 427722 10.1002/1097-0142(197903)43:3<810::aid-cncr2820430306>3.0.co;2-b

[10] Sakamoto A , Kasai N , Sugano H . Poorly differentiated carcinoma of the thyroid. A clinicopathologic entity for a high‐risk group of papillary and follicular carcinomas. Cancer. 1983;52(10):1849‐1855. 6313176 10.1002/1097-0142(19831115)52:10<1849::aid-cncr2820521015>3.0.co;2-x

[11] Howlader N , Noone A , Krapcho M , et al. SEER Cancer Statistics Review. Vol 1975. National Cancer Institute; 2008.

[12] Seyed‐Alagheband S , Sharifian M , Adeli O‐A , Sohooli M , Shekouhi R . Primary synovial sarcoma of thyroid gland: a case report and review of literature. Int J Surg Case Rep. 2021;85:106245. 34330070 10.1016/j.ijscr.2021.106245 PMC8329501

[13] Seyed‐Alagheband S , Shahmoradi M , Adeli O‐A , Shamsi T , Sohooli M , Shekouhi R . Follicular dendritic cell sarcoma of the thyroid gland in a patient with preexisting hashimoto's thyroiditis: a rare case report with a literature review. Case Rep Oncol. 2021;14:1698‐1705. 35082628 10.1159/000520485 PMC8739858

[14] Motazedian M , Shafiei B , Vatankhah P , et al. Differentiated thyroid carcinoma: comparison of histopathologic characteristics, clinical course, and outcome between young children and adolescents. Med Oncol. 2013;30(2):506. 23423788 10.1007/s12032-013-0506-y

[15] Durante C , Costante G , Lucisano G , et al. The natural history of benign thyroid nodules. JAMA. 2015;313(9):926‐935. 25734734 10.1001/jama.2015.0956

[16] Filetti S , Durante C , Torlontano M . Nonsurgical approaches to the management of thyroid nodules. Nat Clin Pract Endocrinol Metab. 2006;2(7):384‐394. 16932321 10.1038/ncpendmet0215

[17] Haugen BR , Alexander EK , Bible KC , et al. 2015 American Thyroid Association management guidelines for adult patients with thyroid nodules and differentiated thyroid cancer: the American Thyroid Association guidelines task force on thyroid nodules and differentiated thyroid cancer. Thyroid. 2016;26(1):1‐133. 26462967 10.1089/thy.2015.0020 PMC4739132

[18] Kaul P , Kaul P , Poonia DR , Jakhetiya A , Arora V , Garg PK . Risk benefit analysis of routine thymectomy for differentiated thyroid cancers: a systematic review. Surg J. 2021;07(04):e307‐e313. 10.1055/s-0041-1736669 PMC8674089 34926812

[19] Carty SE , Cooper DS , Doherty GM , et al. Consensus statement on the terminology and classification of central neck dissection for thyroid cancer: the American thyroid association surgery working group with participation from the American Association of Endocrine Surgeons, American Academy of Otolaryngology—Head and Neck surgery, and American Head and Neck Society. Thyroid. 2009;19(11):1153‐1158. 19860578 10.1089/thy.2009.0159

[20] Akbari M , Abachizadeh K , Khayamzadeh M , Tabatabaee M , Esnaashari F , Motlagh A . Iran cancer report. Cancer Research Center, Shahid Beheshti University of Medical Sciences, Tehran, Qom: Darolfekr. 2008.

[21] Haghpanah V , Soliemanpour B , Heshmat R , et al. Endocrine cancer in Iran: based on cancer registry system. Indian J Cancer. 2006;43(2):80. 16790945 10.4103/0019-509x.25889

[22] Farzadfar F , Peykari N , Larijani B , Rahimzadeh S , Rezaei‐Darzi E , Saeedi Moghaddam S . A comprehensive study on national and sub national trend in thyroid cancer prevalence in the Iranian population, 1990–2010. Iranian J Diabetes Metabolism. 2016;15(2):91‐100.

[23] Edge SB , Edge SB . AJCC Cancer Staging Manual. 8th ed. Springer; 2017.

[24] Wiltshire JJ , Drake TM , Uttley L , Balasubramanian SP . Systematic review of trends in the incidence rates of thyroid cancer. Thyroid. 2016;26(11):1541‐1552. 27571228 10.1089/thy.2016.0100

[25] Do BA , Payne RJ , Bastianelli M , et al. Is age associated with risk of malignancy in thyroid cancer? Otolaryngol–Head Neck Surg. 2014;151(5):746‐750. 25151485 10.1177/0194599814547503

[26] Kwong N , Medici M , Angell TE , et al. The influence of patient age on thyroid nodule formation, multinodularity, and thyroid cancer risk. J Clin Endocrinol Metab. 2015;100(12):4434‐4440. 26465395 10.1210/jc.2015-3100 PMC4667162

[27] Ramezani M , Saeidi M , Zarei A , Hasani M . Investigating the demographic characteristics and pathological manifestations of thyroid cancer during the last two decades (1997–2017) in patients referred to Baqiyatallah hospital, Tehran, Iran. J Diabetes Metab Disord. 2020;19(2):1165‐1172. 33553021 10.1007/s40200-020-00617-x PMC7843909

[28] Wang LL , Li HQ , Chang QG , Li S , Yin DT . [Clinical pathology and incidence trend of thyroid cancer based on 21 980 cases]. Zhonghua Yi Xue Za Zhi. 2020;100(14):1072‐1076. 32294869 10.3760/cma.j.cn112137-20190905-01972

[29] Machens A , Hauptmann S , Dralle H . Disparities between male and female patients with thyroid cancers: sex difference or gender divide? Clin Endocrinol. 2006;65(4):500‐505. 10.1111/j.1365-2265.2006.02623.x 16984243

[30] Machens A , Holzhausen HJ , Dralle H . The prognostic value of primary tumor size in papillary and follicular thyroid carcinoma: a comparative analysis. Cancer. 2005;103(11):2269‐2273. 15856429 10.1002/cncr.21055

[31] Hassan I , Danila R , Aljabri H , et al. Is rapid preparation for thyroidectomy in severe Graves' disease beneficial? The relationship between clinical and immunohistochemical aspects. Endocrine. 2008;33(2):189‐195. 18493879 10.1007/s12020-008-9076-8

[32] Lin D , Qu N , Shi R , Lu Z , Ji Q , Wu W . Risk prediction and clinical model building for lymph node metastasis in papillary thyroid microcarcinoma. Onco Targets Ther. 2016;9:5307‐5316. 27601922 10.2147/OTT.S107913 PMC5004998

[33] Margolick J , Chen W , Wiseman SM . Systematic review and meta‐analysis of unplanned reoperations, emergency department visits and hospital readmission after thyroidectomy. Thyroid. 2018;28(5):624‐638. 29587583 10.1089/thy.2017.0543

[34] Kwak HY , Dionigi G , Liu X , et al. Predictive factors for longer operative times for thyroidectomy. Asian J Surg. 2017;40(2):139‐144. 26321156 10.1016/j.asjsur.2015.07.008

[35] Weiss A , Lee KC , Brumund KT , Chang DC , Bouvet M . Risk factors for hematoma after thyroidectomy: results from the nationwide inpatient sample. Surgery. 2014;156(2):399‐404. 24947642 10.1016/j.surg.2014.03.015

[36] Suzuki S , Yasunaga H , Matsui H , Fushimi K , Saito Y , Yamasoba T . Factors associated with neck hematoma after thyroidectomy: a retrospective analysis using a Japanese inpatient database. Medicine. 2016;95(7):e2812. 26886632 10.1097/MD.0000000000002812 PMC4998632

[37] Terris DJ , Snyder S , Carneiro‐Pla D , et al. American Thyroid Association statement on outpatient thyroidectomy. Thyroid. 2013;23(10):1193‐1202. 23742254 10.1089/thy.2013.0049

[38] Wu TJ , Ha PK , El‐Sayed IH , et al. Socioeconomic disparities in a population of patients undergoing total thyroidectomy for benign disease. Head Neck. 2019;41(3):715‐721. 30521675 10.1002/hed.25421

[39] Papini E , Guglielmi R , Bianchini A , et al. Risk of malignancy in nonpalpable thyroid nodules: predictive value of ultrasound and color‐Doppler features. J Clin Endocrinol Metab. 2002;87(5):1941‐1946. 11994321 10.1210/jcem.87.5.8504

[40] Albuja‐Cruz MB , Goldfarb M , Gondek SS , Allan BJ , Lew JI . Reliability of fine‐needle aspiration for thyroid nodules greater than or equal to 4 cm. J Surg Res. 2013;181(1):6‐10. 23428179 10.1016/j.jss.2012.06.030

[41] McHenry CR , Huh ES , Machekano RN . Is nodule size an independent predictor of thyroid malignancy? Surgery. 2008;144(6):1062‐1069. 19041019 10.1016/j.surg.2008.07.021

[42] Tang JZe , Chua JME , Woon TK , Tan BS , Kiong KL. Large thyroid nodules: should size alone matter? Eur Archiv Oto‐Rhino‐Laryngol. 2022;279(6):3139‐3146. 10.1007/s00405-021-07151-3 34739578

[43] Shin JJ , Caragacianu D , Randolph GW . Impact of thyroid nodule size on prevalence and post‐test probability of malignancy: a systematic review. Laryngoscope. 2015;125(1):263‐272. 24965892 10.1002/lary.24784

[44] Yagmur Y , Akbulut S , Sakarya H , Sogutcu N , Gumus S . Assessment of the relationship between clinical and histopathological features in cases of thyroidectomy. Ann Ital Chir. 2018;89(3):199‐205. 30588916

[45] Sezer A , Celik M , Bulbul BY , et al. Relationship between lymphovascular invasion and clinicopathological features of papillary thyroid carcinoma. Bosn J Basic Med Sci. 2017;17(2):144. 28284178 10.17305/bjbms.2017.1924 PMC5474108

[46] Kim JM , Kim TY , Kim WB , et al. Lymphovascular invasion is associated with lateral cervical lymph node metastasis in papillary thyroid carcinoma. Laryngoscope. 2006;116(11):2081‐2085. 17075398 10.1097/01.mlg.0000242118.79647.a9

[47] Pontius LN , Youngwirth LM , Thomas SM , Scheri RP , Roman SA , Sosa JA . Lymphovascular invasion is associated with survival for papillary thyroid cancer. Endocr Relat Cancer. 2016;23(7):555‐562. 27317633 10.1530/ERC-16-0123

[48] Falvo L , Catania A , D'Andrea V , Marzullo A , Giustiniani MC , De Antoni E . Prognostic importance of histologic vascular invasion in papillary thyroid carcinoma. Ann Surg. 2005;241(4):640‐646. 15798466 10.1097/01.sla.0000157317.60536.08 PMC1357068

[49] Rubinstein JC , Dinauer C , Herrick‐Reynolds K , Morotti R , Callender GG , Christison‐Lagay ER . Lymph node ratio predicts recurrence in pediatric papillary thyroid cancer. J Pediatr Surg. 2019;54(1):129‐132. 30361076 10.1016/j.jpedsurg.2018.10.010

[50] Rozenblat T , Hirsch D , Robenshtok E , et al. The prognostic value of lymph node ratio in medullary thyroid carcinoma: a multi‐center study. Eur J Surg Oncol. 2020;46(11):2023‐2028. 32389525 10.1016/j.ejso.2020.04.016

[51] Lee SG , Ho J , Choi JB , et al. Optimal cut‐off values of lymph node ratio predicting recurrence in papillary thyroid cancer. Medicine. 2016;95(5):e2692. 26844509 10.1097/MD.0000000000002692 PMC4748926

[52] Alnuaimi AM , Alameri M , Mustafa H , Khalil AB . MON‐507 a descriptive study of clinical and surgical characteristics of patients with thyroid cancer: a 10‐year retrospective study from UAE. J Endocr Soc. 2020;4(Suppl 1):MON‐507. 10.1210/jendso/bvaa046.1464

[53] Ghadhban BR , kadam SM , sultan HA . Incidence of differentiated thyroid carcinoma in multinodular goiter patients. Int J Surg Open. 2018;15:18‐24.

[54] Mincer DL , Jialal I . Hashimoto thyroiditis. 2017. 29083758

[55] Nasiri S , Yazd SMM , Gholami M , Shahriarirad S , Sharghi S , Shahriarirad R . The evaluation of locoregional tumoral involvement in the cooccurrence of hashimoto thyroiditis with papillary thyroid cancer: a case controlled study. BMC Endocr Disord. 2023;23(1):66. 36964545 10.1186/s12902-023-01322-5 PMC10037788

[56] Miranda‐Filho A , Lortet‐Tieulent J , Bray F , et al. Thyroid cancer incidence trends by histology in 25 countries: a population‐based study. Lancet Diabetes Endocrinol. 2021;9(4):225‐234. 33662333 10.1016/S2213-8587(21)00027-9

[57] Ahn HS , Welch HG . South Korea's thyroid‐cancer “epidemic”—turning the tide. N Engl J Med. 2015;373(24):2389‐2390. 26650173 10.1056/NEJMc1507622

[58] Bibbins‐Domingo K , Grossman DC , Curry SJ , et al. Screening for thyroid cancer: US Preventive Services Task Force recommendation statement. JAMA. 2017;317(18):1882‐1887. 28492905 10.1001/jama.2017.4011

[59] Davies L , Morris L . The USPSTF recommendation on thyroid cancer screening: don't “check your neck”. JAMA Otolaryngol‐Head Neck Surg. 2017;143(8):755‐756. 28492875 10.1001/jamaoto.2017.0502

[60] Roman BR , Morris LG , Davies L . The thyroid cancer epidemic, 2017 perspective. Curr Op Endocrinol, Diabetes Obesity. 2017;24(5):332‐336. 10.1097/MED.0000000000000359 PMC5864110 28692457

[61] Zhang C , Li Y , Li J , Chen X . Total thyroidectomy versus lobectomy for papillary thyroid cancer: a systematic review and meta‐analysis. Medicine. 2020;99(6):e19073. 32028431 10.1097/MD.0000000000019073 PMC7015547

[62] Zheng W , Li J , Lv P , Chen Z , Fan P . Treatment efficacy between total thyroidectomy and lobectomy for patients with papillary thyroid microcarcinoma: a systemic review and meta‐analysis. Eur J Surg Oncol. 2018;44(11):1679‐1684. 30158063 10.1016/j.ejso.2018.08.004

[63] Barczyński M , Konturek A , Hubalewska‐Dydejczyk A , Gołkowski F , Nowak W . Ten‐year follow‐up of a randomized clinical trial of total thyroidectomy versus Dunhill operation versus bilateral subtotal thyroidectomy for multinodular non‐toxic goiter. World J Surg. 2018;42(2):384‐392. 28942461 10.1007/s00268-017-4230-1 PMC5762805

[64] Molteni G , Bonali M , Mattioli F , et al. Central compartment revision surgery for persistent or recurrent thyroid carcinoma: analysis of survival and complication rate. Eur Arch Otrhinolaryngol. 2019;276(2):551‐557. 10.1007/s00405-018-5239-2 30535975

[65] Chen L , Wu Y‐H , Lee C‐H , Chen H‐A , Loh E‐W , Tam K‐W . Prophylactic central neck dissection for papillary thyroid carcinoma with clinically uninvolved central neck lymph nodes: a systematic review and meta‐analysis. World J Surg. 2018;42(9):2846‐2857. 29488066 10.1007/s00268-018-4547-4

[66] Su H , Li Y . Prophylactic central neck dissection and local recurrence in papillary thyroid microcarcinoma: a meta‐analysis. Brazilian J Otorhinolaryngol. 2019;85:237‐243. 10.1016/j.bjorl.2018.05.004 PMC9452245 30017872

[67] Zaydfudim V , Feurer ID , Griffin MR , Phay JE . The impact of lymph node involvement on survival in patients with papillary and follicular thyroid carcinoma. Surgery. 2008;144(6):1070‐1078. 19041020 10.1016/j.surg.2008.08.034

[68] Lundgren CI , Hall P , Dickman PW , Zedenius J . Clinically significant prognostic factors for differentiated thyroid carcinoma: a population‐based, nested case–control study. Cancer. 2006;106(3):524‐531. 16369995 10.1002/cncr.21653

[69] Shahriarirad R , Meshkati Yazd SM , Zahedi R , Mokhtari Ardekani A , Rekabi MM , Nasiri S . Evaluation of the role of prophylactic bilateral central neck lymph node dissection in patients with papillary thyroid carcinoma: a case controlled study. Updates Surg. 2023;75(3):679‐689. 36527603 10.1007/s13304-022-01440-0

[70] Alibakhshi A , Sheikhi S , Meshkati Yazd SM , Ardekani A , Ranjbar K , Shahriarirad R . The incidence and features of Delphian lymph node involvement in patients with papillary thyroid carcinoma. BMC Surg. 2022;22(1):320. 35987629 10.1186/s12893-022-01742-5 PMC9392353

[71] Stulak JM . Value of preoperative ultrasonography in the surgical management of initial and reoperative papillary thyroid cancer. Arch Surg. 2006;141(5):489‐496. 16702521 10.1001/archsurg.141.5.489

[72] Lim H , Devesa SS , Sosa JA , Check D , Kitahara CM . Trends in thyroid cancer incidence and mortality in the United States, 1974‐2013. JAMA. 2017;317(13):1338‐1348. 28362912 10.1001/jama.2017.2719 PMC8216772

[73] Kloos (Chair) RT , Eng C , Evans DB , et al. Medullary thyroid cancer: management guidelines of the American Thyroid Association. Thyroid. 2009;19(6):565‐612. 19469690 10.1089/thy.2008.0403

[74] Hauch A , Al‐Qurayshi Z , Randolph G , Kandil E . Total thyroidectomy is associated with increased risk of complications for low‐and high‐volume surgeons. Ann Surg Oncol. 2014;21(12):3844‐3852. 24943236 10.1245/s10434-014-3846-8

[75] Caron NR , Clark OH . Papillary thyroid cancer: surgical management of lymph node metastases. Curr Treat Options Oncol. 2005;6(4):311‐322. 15967084 10.1007/s11864-005-0035-9

[76] Gršić K . Prophylactic central neck dissection in well‐differentiated thyroid cancer. Acta Clinica Croatica. 2020;59(suppl 1):87. 34219889 10.20471/acc.2020.59.s1.11 PMC8212603

[77] Shahriarirad R , Meshkati Yazd SM , Ardekani A , Mokhtari Ardekani A , Moradi N , Nasiri S . Calcitriol supplementation before parathyroidectomy and calcium level after surgery in parathyroid adenoma patients: a randomized controlled trial. J Endocrinol Invest. 2023;46(5):985‐990. 10.1007/s40618-022-01963-8 36459369

[78] Li W , Wang B , Jiang Z , Feng Y , Zhang W , Qiu M . The role of thymus preservation in parathyroid gland function and surgical completeness after bilateral central lymph node dissection for papillary thyroid cancer: a randomized controlled study. Int J Surg. 2019;65:1‐6. 30818068 10.1016/j.ijsu.2019.02.013

[79] Nasiri S , Meshkati Yazd SM , Mokhtari Ardekani A , Fazlollahpour‐Naghibi A , Shahintaj M , Shahriarirad R . The effect of thymectomy during central neck dissection in papillary thyroid carcinoma: a case‐controlled study. Updates Surg. 2023;75(1):227‐233. 10.1007/s13304-022-01428-w 36436160

